# Reduced serum sestrin 2 levels in Hashimoto’s disease: a cross-sectional study on a potential pathophysiological and diagnostic role

**DOI:** 10.1530/EC-25-0546

**Published:** 2025-09-29

**Authors:** Merve Ates, Mesut Ates, Murat Alisik, Ozgur Mehmet Yis

**Affiliations:** ^1^ Department of Medical Biochemistry, Bolu Public Health Laboratory, Bolu, Turkey; ^2^ Department of Internal Disease, BAIBU, Izzet Baysal Training and Research Hospital, Bolu, Turkey; ^3^ Department of Medical Biochemistry, BAIBU Faculty of Medicine, Bolu, Turkey

**Keywords:** Hashimoto, hypothyroidism, mTOR, sestrin 2, thyroiditis

## Abstract

**Objective:**

Expression of mammalian target of rapamycin (mTOR), which plays a key role in coordinating the balance between cell growth and autophagy, is elevated in thyroid tissues of patients with Hashimoto’s disease. Sestrins are an evolutionarily conserved, stress-inducible protein family that reduces oxidative stress and regulates the adenosine monophosphate-dependent protein kinase (AMPK)-mTOR signaling pathway. The aim of this study was to investigate the potential role and significance of sestrin 2 (SESN2), a member of the sestrin family, in Hashimoto’s disease.

**Methods:**

This cross-sectional study included patients with Hashimoto’s disease and healthy volunteers. Thyroid autoantibodies, free T_4_, TSH, and sestrin 2 were measured from blood samples. Sestrin 2 was analyzed by an ELISA kit.

**Results:**

One hundred ten patients and 64 healthy volunteers were included in the study. Median SESN2 levels in the patient group (1.36 ng/mL (1.10–2.03)) were significantly lower than in the control group (1.83 ng/mL (1.34–2.64)) (*P* = 0.002). There was no significant difference in SESN2 levels between euthyroid and subclinical hypothyroidism subgroups of patients (*P* > 0.05).

**Conclusion:**

In this study, serum SESN2 levels were found to be lower in patients with Hashimoto’s disease than in healthy adults, suggesting that SESN2 may have a role in the pathophysiology of Hashimoto’s disease. These results indicate that further research is needed to better understand the effect of regulation of SESN2 in Hashimoto’s disease on the course and pathological processes of the disease.

## Introduction

Hashimoto’s disease is a chronic autoimmune disease in which the thyroid gland is destroyed by thyroid-specific autoantibodies and cellular immunity (1). One of the factors involved in the pathogenesis of Hashimoto’s disease is insufficient autophagy seen in thyroid tissue. The mammalian target of rapamycin (mTOR), a protein kinase that controls the balance between autophagy and cell growth, is inhibited to initiate autophagy when cells are deprived of nutrients. In Hashimoto’s disease, the expression level of mTOR protein in thyroid tissue is higher than in normal thyroid tissue, and therefore less autophagy occurs in thyroid tissue (2).

Sestrins are an evolutionarily conserved protein family whose expression increases in conditions such as DNA damage, stress, oxidative stress, and inflammation (3). Sestrin proteins have important physiological functions in inflammation, fibrosis, cell damage, and metabolism (4). The sestrin family consists of three groups: SESN1, SESN2, and SESN3. While all sestrin proteins are induced by oxidative, genotoxic, and metabolic stressors, only SESN2 is induced by stressors such as hypoxia, endoplasmic reticulum stress (ERS), mitochondrial stress, and starvation (5). The SESN2 protein activates AMP-sensitive protein kinase (AMPK), phosphorylates tuberous sclerosis complex protein (TSC2), stimulates GAP activity, and ultimately inhibits mTOR (6). SESN2 is a leucine sensor for the mTOR complex 1 (mTORC1) pathway. The leucine-interacting SESN2 protein acts on the GATOR2-GATOR1-Rag axis, the major signaling pathway regulating mTORC1 activation, to inhibit mTORC1 (7). Another way in which SESN2 regulates autophagy via mTOR is by modulating the mTORC1/AMPK pathway via AMPK, an important nutrient sensor that regulates metabolic energy homeostasis (6, 8). Oxidative stress induces SESN2 expression, and SESN2 acts as a defense regulator against excessive oxidative stress to protect cells through nuclear factor erythroid 2-related factor 2 (Nrf2) and c-Jun N-terminal kinase-activator protein-1 (JNK-AP-1) (9, 10). Activating transcription factor 6 (ATF6) regulates SESN2 expression, and overexpression of SESN2 reduces ERS-associated cell death, while suppression of SESN2 exacerbates ERS, resulting in increased ERS-mediated cell apoptosis (11).

There are studies suggesting that Hashimoto’s disease may increase the risk of papillary thyroid cancer (PTC) (12, 13). In a study on PTC treatment, it was shown that SESN2 levels were lower in cancerous tissues compared to normal thyroid tissues (14). The known relationship between SESN2 and mTOR suggests that the high mTOR levels observed in Hashimoto’s disease may be due to low SESN2 levels in patients, as in PTC. In this context, the potential role of SESN2 in Hashimoto’s disease may contribute to both the understanding of the pathophysiology of the disease and the development of new treatment approaches. The aim of this study was to investigate the potential role and importance of SESN2 in Hashimoto’s disease.

## Materials and methods

### Study design and participants

This cross-sectional study was conducted between July 2023 and March 2024 with 110 patients diagnosed with Hashimoto’s disease or being followed for Hashimoto’s disease, and 64 healthy adult volunteers with no known disease or physical findings who presented for a general health examination to our hospital’s Internal Medicine Clinic. Individuals with heart failure, a history of malignancy, a history of acute infection, chronic liver disease, concomitant thyroid disease, pregnancy, a history of acute coronary syndrome in the last 6 months, or end-stage renal disease were not included in this study. Patients aged 18 and over and under 65 who met these criteria were included in the study. Demographic data for Hashimoto’s patients and controls were recorded upon admission to the clinic. The patient group was divided into two groups: euthyroid and subclinical hypothyroid based on thyroid function tests. Patients with elevated TSH values and normal fT_4_ values were included in the subclinical hypothyroidism group, while patients with normal TSH and fT_4_ values were included in the euthyroidism group.

### Laboratory testing

Serum TSH (mIU/mL), fT_4_ (ng/dL), anti-TPO (kIU/L), and anti-TG (kIU/L) values were measured by chemiluminescent microparticle immunoassay (CMIA) method on the Architect i2000 (Abbott, USA) autoanalyzer. The remaining serum samples were aliquoted into Eppendorf tubes and stored at −80°C for the determination of human SESN2 levels. Upon completion of sample collection, the samples were simultaneously brought to room temperature and analyzed with the SESN2 ELISA kit. Human SESN2 (sestrin 2) ELISA Kit (BT LAB, China, sensitivity 0.01 ng/mL, detection range: 0.05–15 ng/mL, code: E3437Hu) was used for ELISA measurement. Intra-assay CVs stated by the manufacturer are: CV1%: 5.9, CV2%: 2.8, CV3%: 4.9.

### Statistical methods

Data analysis was performed using SPSS 22.0 program (IBM Corp. Released 2013. IBM SPSS Statistics for Windows, Version 22.0., USA). The normality of numerical data was assessed using the Kolmogorov–Smirnov test and distribution histograms. For the comparison of numerical variables, the independent samples t-test was used for two-group comparisons of normally distributed data, with results presented as mean ± standard deviation, while the Mann–Whitney U test was applied for two-group comparisons of non-normally distributed data, with results expressed as median (1st–3rd quartile). For comparisons of non-normally distributed data involving more than two groups, the Kruskal–Wallis test was used, followed by the post-hoc Dunn-Bonferroni pairwise comparison test when necessary. Categorical variables were compared using the Pearson chi-square test. Correlation analysis was performed using the Pearson correlation test for parametric numerical variables and the Spearman correlation test for nonparametric numerical variables. Receiver operating characteristic (ROC) analysis was performed to examine the diagnostic value of SESN2 level, and sensitivity and specificity values were calculated according to the determined cut-off point. Logistic regression analysis was used to determine the predictive values of the parameters on the disease. *P* < 0.05 was considered statistically significant.

## Results

Demographic characteristics, thyroid function tests, autoantibodies, and serum SESN2 levels of the patient and control groups are given in Table 1. There was no significant difference between the groups in terms of age, age groups, and gender (*P* values 0.368; 0.317; 0.896, respectively). Significant differences were found between the groups for TSH, fT_4_, anti-TPO, and anti-TG results (*P* values < 0.001; 0.039; <0.001; <0.001, respectively).

**Table 1:** Demographic data, thyroid function tests, autoantibodies, and serum SESN2 levels of patient and control groups.

		Patients (*n* = 110)	Controls (*n* = 64)	*P*
Age, years	Median (Q1–Q3)	40 (32–49)	38 (33–45)	0.368
	<34 years, *n* (%)	35 (31.8)	22 (34.4)	
	35–44 years, *n* (%)	34 (30.9)	25 (39.1)	
Age, group	45–54 years, *n* (%)	22 (20)	12 (18.8)	0.317
	>55 years, *n* (%)	19 (17.3)	5 (7.8)	
Gender	Female, *n* (%)	100 (90.9)	57 (89.1)	0.896
	Male, *n* (%)	10 (9.1)	7 (10.9)	
TSH (μIU/mL)	Median (Q1–Q3)	2.59 (1.44–4.49)	1.42 (1.02–1.91)	<0.001
Free T_4_ (ng/dL)	Mean ± SD	0.96 ± 0.13	0.92 ± 0.10	0.039
Anti-TPO (kIU/L)	Median (Q1–Q3)	132.67 (11.31–482.11)	0.35 (0.11–0.88)	<0.001
Anti-TG (kIU/L)	Median (Q1–Q3)	25.46 (10.08–154.14)	0.92 (0.69–1.56)	<0.001
SESN2 (ng/mL)	Median (Q1–Q3)	1.36 (1.10–2.03)	1.83 (1.34–2.64)	0.002

Median (Q1–Q3), median (1st–3rd quartile value); mean ± SD, mean ± standard deviation; *n*, number.

The median (Q1 to Q3) values of serum SESN2 levels in the patient group were 1.36 ng/mL (1.10–2.03), which were significantly lower than the median (Q1 to Q3) values of serum SESN2 levels in the control group, 1.83 ng/mL (1.34–2.64) (*P* = 0.002).

The relationship between serum SESN2 level and demographic and clinical characteristics is given in Table 2. A statistically significant difference was found between the serum SESN2 levels for age groups (*P* = 0.002). While 64 patients were treated with only levothyroxine, 46 patients were followed up without treatment. There was no significant relationship between treatment status and SESN2 levels.

**Table 2:** Demographic and clinical evaluation of serum SESN2 levels.

		*n*	SESN2 (ng/mL)	*P*
			Median (Q1–Q3)	
Gender	Female	157	1.45 (1.19–2.32)	0.446
	Male	17	1.42 (1.10–1.73)	
Age, groups	<34	57	1.95 (1.35–4.04)^a^	0.002
	35–44	59	1.46 (1.16–2.07)	
	45–54	34	1.29 (1.05–2.29)^b^	
	>55	24	1.33 (1.06–1.64)^c^	
Subgroup	Euthyroid	83	1.36 (1.08–2.40)^d^	0.007
	Subclinical hypothyroidism	27	1.38 (1.19–1.65)^d^	
	Control	64	1.83 (1.34–2.64)	
Antibody	Anti-TPO positive anti-TG positive	75	1.36 (1.14–2.13)	0.638
	Anti-TPO positive anti-TG negative	14	1.36 (0.77–3.26)	
	Anti-TPO negative anti-TG positive	21	1.35 (1.06–1.47)	
Treatment (levothyroxine)	Yes	64	1.36 (1.08–1.82)	0.734
	No	46	1.37 (1.11–2.22)	

Median (Q1–Q3), median (1st–3rd quartile value); *n*, number. There is a significant difference between a and b, and a and c; d: significantly different from the control group according to the Dunn-Bonferroni pairwise comparison test.

When the serum SESN2 levels of the patient groups were examined, the median (Q1 to Q3) value of serum SESN2 levels of the euthyroid patient group was 1.36 ng/mL (1.08–2.40), and the median (Q1 to Q3) value of serum SESN2 levels of the subclinical hypothyroid patient group was 1.38 ng/mL (1.19–1.65). No statistically significant difference was found between the patient groups in terms of serum SESN2 levels (*P* = 0.975).

**Figure 1:**
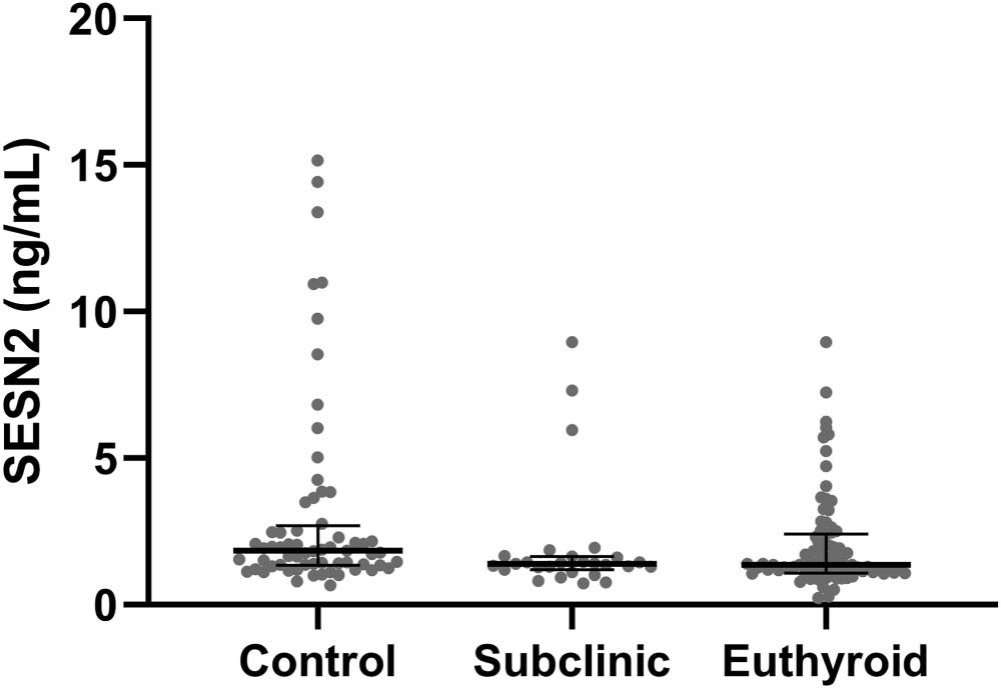
Box plot representation of sestrin 2 levels in Hashimoto’s disease subgroups and control group (median, 1st and 3rd quartile values, minimum and maximum values are shown in the box plot).

A statistically significant difference in SESN2 levels was observed between the patient subgroups and the control group (*P* = 0.007, Kruskal–Wallis test, Fig. 1). Post-hoc analysis revealed a significant difference in SESN2 levels between euthyroid patients and the control group (*P* = 0.006) and between subclinical hypothyroid patients and the control group (*P* = 0.009).

When the patient group was divided into two groups as euthyroid and subclinical hypothyroid according to thyroid function tests, no significant difference was found between the patient groups and the control group in terms of gender and age groups (*P* = 0.851; *P* = 0.630, respectively) (Table 3).

**Table 3:** Demographic and clinical characteristics of patient and control groups.

		Patients		Controls (*n* = 64) (%)	*P*
		Euthyroid (*n* = 83) (%)	Subclinical hypothyroidism (*n* = 27) (%)		
Gender	Female	76 (91.6)	24 (88.9)	57 (89.1)	0.851
	Male	7 (0.4)	3 (13)	7 (10.9)	
Age, years	Median (Q1–Q3)	39 (30–49)	41 (36–50)	38 (33–45)	0.386
Age, groups	<34 years	31 (37.3)	4 (14.8)	22 (34.40)	
	35–44 years	23 (27.7)	11 (40.7)	25 (39.10)	
	45–54 years	13 (15.7)	9 (33.3)	12 (18.8)	0.630
	>55 years	16 (19.3)	3 (11.1)	5 (7.8)	
	Anti-TPO positive anti-TG positive	52 (62.7)	23 (85.2)	0 (0)	
	Anti-TPO positive anti-TG negative	12 (14.5)	2 (7.4)	0 (0)	
Antibody	Anti-TPO negative anti-TG positive	19 (22.9)	2 (7.4)	0 (0)	<0.001
	Ant-TPO negative anti-TG negative	0 (0)	0 (0)	64 (100)	
Treatment	Yes	54 (65.1)	10 (37)	0 (0)	<0.001
	No	29 (34.9)	17 (63)	0 (0)	

*n*, number.

A negative correlation was found between serum SESN2 level and age and anti-TG (rho = −0.285, *P* < 0.001; and rho = −0.214, *P* = 0.005, respectively). There was no significant relationship between serum SESN2 level and TSH, free T_4_, and anti-TPO (rho = −0.017, *P* = 0.826; rho = 0.090, *P* = 0.240; and rho = −0.108, *P* = 0.157, respectively).

**Figure 2:**
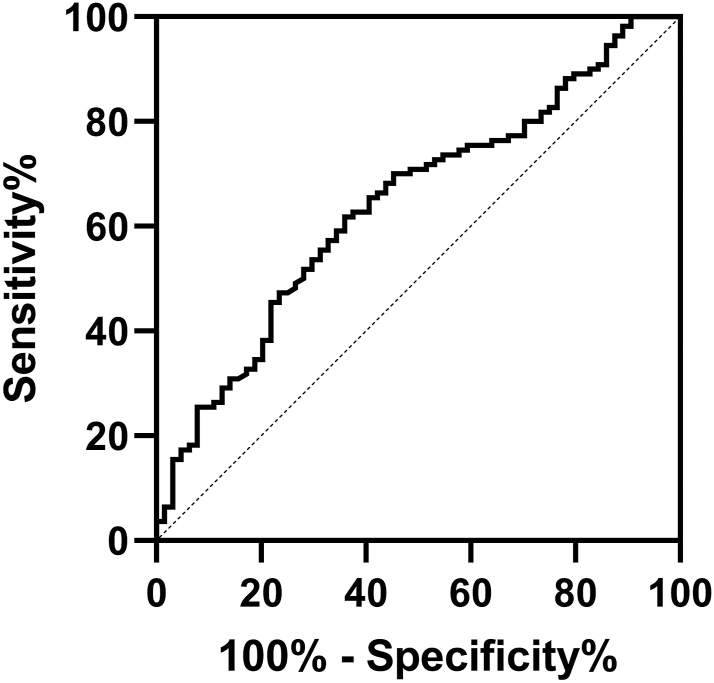
ROC analysis of serum SESN2 level.

In the ROC curve analysis performed to determine how effective a factor serum SESN2 measurements are in distinguishing Hashimoto’s disease patients from the control group, the area under the curve was calculated as 0.644 (95% CI: 0.560–0.727). When the cut-off value for SESN2 was determined as 1.76 ng/mL, 70% sensitivity and 54.7% specificity were detected (Fig. 2).

A logistic regression was performed to ascertain the effects of age, gender, TSH, and SESN2 on the likelihood that participants have Hashimoto’s disease. The logistic regression model was statistically significant (*R*^2^ = 0.279; *P* < 0.001, Nagelkerke *R*^2^ = 0.381). No statistically significant effect was found for age and gender (*P* = 0.652 and *P* = 0.827, respectively). An increase in TSH level increased the risk of developing Hashimoto’s disease by 2.844 times (95% confidence interval: 1.834–4.41, *P* < 0.001), while a decrease in serum SESN2 level increased the risk of the disease by 0.789 times (95% confidence interval: 0.658–0.947, *P* = 0.011).

## Discussion

In this study, it was found that the serum SESN2 levels of patients with Hashimoto’s disease were lower than those of healthy adults. The 70% sensitivity and 54.7% specificity we obtained in the ROC analysis indicate that serum SESN2 level has a modest ability to distinguish Hashimoto patients from healthy adults. There was no significant difference in SESN2 between euthyroid and subclinical hypothyroid patients; however, in both groups, SESN2 was significantly lower than in the control group. This suggests that this decrease may be related to the underlying autoimmune pathophysiology of Hashimoto’s disease rather than impairment of thyroid function. There was no significant association between SESN2 and gender, anti-TPO and/or anti-TG positivity, and treatment status among the study participants, while a significant association was found between SESN2 and age groups. SESN2 levels were higher in participants aged 34 and under than in participants aged 55 and over and in the 45–54 age range. There was a correlation between SESN2 levels and age and anti-TG; however, no correlation was found between SESN2 levels and TSH, fT_4_, and anti-TPO. SESN2 levels were found to decrease with increasing age and anti-TG values increased in the study participants. To the best of our knowledge, serum SESN2 levels have not been previously studied in Hashimoto’s disease.

It has been shown that autophagy regulated by the mTOR signaling pathway is effective in Hashimoto’s disease (2). Peng *et al.* (15) showed that SESN2 activates the AMPK pathway and inhibits mTORC1 to promote autophagy. In our study evaluating SESN2 levels in Hashimoto’s disease, we found that SESN2 levels were lower in patients with Hashimoto’s disease compared to healthy adults. When we interpret this finding in conjunction with previous studies in the literature, we suggest that the decrease in SESN2 levels in Hashimoto’s disease may lead to increased mTOR expression, ultimately resulting in suppressed autophagy.

In a previous study by Qu *et al.* (14), it was shown that a substance called calycosin induces apoptosis and autophagy in human PTC cells through the SESN2/AMPK/mTOR pathway and that SESN2 levels were lower in PTC cells than in normal thyroid tissue. There are many studies on Hashimoto’s disease as a risk factor for PTC (12, 13, 16). Considering the relationship between Hashimoto’s disease and PTC, the low SESN2 levels in PTC cells support the low SESN2 levels in Hashimoto’s disease detected in our study.

Zhan *et al.* (17) showed that the SESN/AMPK/mTOR pathway is also effective in another type of thyroid cancer, medullary thyroid cancer (MTC). They found that a new antineoplastic compound called 2-imino-6-methoxy-2H-chromone-3-carbothioamide (IMCA), which targets the MTC NR4A1 (orphan nuclear receptor 4A1), triggered tumor cell death in a dose-dependent manner. It is thought that IMCA may be a specific antagonist of NR4A1 through the NR4A1 and SESN/AMPK/mTOR signaling pathway and therefore can be used in the treatment of thyroid carcinomas. Considering this study on the treatment of MTC, it is thought that studies evaluating new treatments targeting the SESN/AMPK/mTOR pathway in Hashimoto’s disease are needed.

Considering the properties of sestrins, such as activating AMPK, suppressing mTORC1, inducing autophagy, and being antioxidants, it is thought that they may contribute to delaying aging and suppressing age-related diseases (18). In a study conducted by Lee *et al.* by creating an SESN deficiency model in Drosophila flies and mice, it was shown that endogenous SESN activity is necessary to prevent various age-related pathologies (19). In another study, Yang *et al.* showed that C. elegans with SESN deficiency had shorter lifespans, were hypersensitive to oxidative stress, and showed premature aging in muscle (20). When the relationship between SESN2 levels and age was evaluated in our study, it was found that SESN2 levels decreased as age increased. It is thought that this situation may be the result of increased oxidative stress and decreased autophagy in the body as age increases.

Another study in the literature on SESN2 is the study conducted by Odabaş *et al.*, in which SESN2 levels were evaluated in patients with multiple sclerosis (MS) (21). Accumulation of inflammatory cells in the central nervous system is a critical step in the development of demyelination in MS (22). Similarly, in Hashimoto’s disease, also known as chronic lymphocytic thyroiditis, lymphocyte infiltration in the thyroid parenchyma is one of the most important pathogenesis mechanisms (1). In addition, as in Hashimoto’s disease, apoptosis and oxidative stress have been shown to be effective in the etiology of MS (22). In this study on MS, SESN2 levels were found to be low, as we found in Hashimoto’s disease.

This study evaluated euthyroid and subclinical hypothyroid patients with Hashimoto’s disease, and one of the limitations of the study was that patients with significant hypothyroidism were not included. The relatively low sample size of 110 patients and 64 healthy volunteers, the inability to evaluate the histological data of the patients, and the mTOR signaling pathway of all participants are also limitations of the study. It is thought that this study may be a pioneer for *in vitro* studies that can be conducted using thyroid tissue in the future.

## Conclusion

To the best of our knowledge, no study to date has evaluated serum SESN2 levels in patients with Hashimoto’s disease; this investigation is the first to examine the potential diagnostic or pathophysiological significance of SESN2 in Hashimoto’s disease. Considering that SESN2 is an important protein that regulates autophagy and suppresses the mTOR signaling pathway, the low level of SESN2 in serum and in thyroid cells suggests that it is one of the mechanisms of development of Hashimoto’s disease, which has insufficient autophagy in its pathogenesis. Longitudinal evaluation of the role of SESN2 in Hashimoto’s disease and investigation of new treatment approaches targeting this protein are considered important areas for future studies. At the same time, it is thought that detailed examination of the effect of SESN2 levels on the development of thyroid cancer may be useful both in terms of disease prognosis and the development of new treatment strategies.

The use of SESN2 in the diagnosis, staging, and evaluation of response to treatment in Hashimoto’s disease will be valuable in identifying patients before clinical symptoms begin, improving their quality of life, initiating timely and effective treatment, evaluating the response, and even avoiding unnecessary treatments.

## Declaration of interest

The authors declare that there is no conflict of interest that could be perceived as prejudicing the impartiality of the work reported.

## Funding

This work did not receive any specific grant from any funding agency in the public, commercial, or not-for-profit sector.

## Author contribution statement

Merve Ates was responsible for conceptualization, data curation, formal analysis, funding acquisition, investigation, methodology, project administration, validation, visualization, and writing the original draft, writing review and editing. Mesut Ates was responsible for data curation, formal analysis, investigation, methodology, software, and writing the original draft. Murat Alisik contributed to conceptualization, funding acquisition, methodology, project administration, supervision, resources, and validation. Ozgur Mehmet Yis contributed to methodology, validation, formal analysis, resources, and supervision.

## Statement of ethics

This prospective cross-sectional study was approved by Bolu Abant Izzet Baysal University Clinical Research Ethics Committee (approval No. 2023/239). Subjects have given their written informed consent for this study.
